# Diversity of *Clostridium perfringens* toxin-genotypes from dairy farms

**DOI:** 10.1186/s12866-016-0812-6

**Published:** 2016-08-30

**Authors:** Svenja Fohler, Guenter Klein, Martina Hoedemaker, Theresa Scheu, Christian Seyboldt, Amely Campe, Katharina Charlotte Jensen, Amir Abdulmawjood

**Affiliations:** 1 Institute of Food Quality and Food Safety, Research Center for Emerging Infections and Zoonoses, University of Veterinary Medicine Hannover, Foundation, Hannover, Germany; 2 Clinic for Cattle, University of Veterinary Medicine Hannover, Foundation, Hannover, Germany; 3 Friedrich-Loeffler-Institut, Institute of Bacterial Infections and Zoonoses, Jena, Germany; 4 Department of Biometry, Epidemiology and Information Processing, University of Veterinary Medicine Hannover, Foundation, Hannover, Germany

**Keywords:** *C. perfringens*, Dairy farms, Cattle, Toxin-genotype, Rumen content

## Abstract

**Background:**

*Clostridium* (*C.*) *perfringens* is the causative agent of several diseases in animals and humans, including histotoxic and enteric infections. To gain more insight into the occurrence of its different toxin-genotypes in dairy herds, including those toxin genes previously associated with diseases in cattle or humans, 662 isolates cultivated from feces, rumen content and feed collected from 139 dairy farms were characterized by PCR (detecting *cpa*, *cpb*, *iap*, *etx, cpe,* and both allelic variants of *cpb2*).

**Results:**

Isolates from feces were assigned to type A (*cpa* positive, *n* = 442) and D (*cpa* and *etx* positive, *n* = 2). Those from rumen content (*n* = 207) and feed (*n* = 13) were all assigned to type A. The consensus and atypical variants of the *cpb2* gene were detected in 64 (14.5 %) and 138 (31.22 %) of all isolates from feces, and 30 (14.5 %) and 54 (26.1 %) of all isolates from rumen content, respectively.

**Conclusion:**

Both allelic variants of *cpb2* occurred frequently in animals without signs of acute enteric disease, whereby the atypical variant dominated. Five (0.8 %) of all type A isolates were positive for the *cpe* gene*.* Therefore, the present study indicates that dairy cows are no primary source for potentially human pathogenic enterotoxin gene positive strains.

## Background

*Clostridium* (*C*.) *perfringens* is the primary cause of a range of diseases in animals and humans [1]. Most important are gas gangrene, which occurs mainly after contamination of wounds with cells or spores [2], and enteric diseases that either solely affect the gut or in addition induce generalized symptoms, e.g. pulpy kidney disease in ruminants [3]. It is classified into five types, A to E, depending on the production of four major toxins (alpha, beta, epsilon, and iota) [3, 4]. Distinct types of these are associated with certain diseases. In addition, clinical importance is attributed to the beta-2 and the enterotoxin, two of up to 12 potentially occurring minor toxins [5].

The alpha toxin is the major pathogenicity factor in gas gangrene. The coding gene (*cpa*) is chromosomally-located, highly-conserved, and therefore present in all *C. perfringens* strains [6]. The second major toxin that is coded by *cpb* is the beta toxin. It is involved in necrotic enteritis in several species, including cattle, and enterotoxaemia like lamb dysentery [4]. The third major toxin, the epsilon toxin coded by *etx*, is important for enterotoxaemia in sheep, goats, and less frequently in cattle [4]. Type E that produces the iota toxin, which consists of two components coded by *iap* and *ibp*, was previously associated with enterotoxaemia in calves as well as in lambs and rabbits [7].

The role of the beta-2 toxin in intestinal diseases in different animal species is still not entirely clear [4]. Until now, it has been demonstrated to be a key factor for enteritis in piglets [8, 9]. The reported frequencies for cattle and the conclusions drawn from different studies concerning its role, especially in enteric disease cases, differ widely [10]. It is coded by the plasmid-borne *cpb2* that occurs in two variants (consensus and atypical) [11]. The most important minor toxin for human intestinal diseases is the enterotoxin. This toxin is coded by *cpe* that can be located on the chromosome or on a plasmid. It causes food poisoning, antibiotic-associated diarrhea, and sporadic diarrhea [12]. In animals it was previously suspected of being associated with enteritis in several species, but its importance still remains unclear [5, 13]. *C. perfringens* was also suspected of being involved in the development of additional diseases, including an enterotoxaemia in calves as well as an acute necro-hemorrhagic enteritis in adult cattle [14, 15].

Till now studies on the diversity of *C. perfringens* types in mature dairy cattle testing for the coding genes of all above mentioned toxins are rare. Thus, within this study *C. perfringens* isolates, cultured from samples collected from a large number of dairy farms, were characterized by testing with a multiplex PCR for the four major toxin genes (*cpa*, *cpb*, *iap*, and *etx*) as well as the presence of the *cpb2* (consensus and atypical variant) and *cpe*.

## Methods

### Sample collection

Fecal samples (*n* = 1388) from the rectum and rumen content (*n* = 1389) were collected from each of ten lactating cows from 139 dairy farms in Germany within a study examining the occurrence of *Clostridium* species in dairy herds. The study population was previously described by Seyboldt et al. [16]. Feces and rumen content were transferred into 120 and 50 ml screw cap tubes, respectively. The texture was documented for each fecal sample. Additionally, 410 feed samples were collected (up to five per farm depending on the fed ration). All samples were immediately cooled at < 8 °C.

### Culture conditions

Anaerobic cultivation of *Clostridium* isolates was performed at the Institute for Microbiology, Department of Infectious Diseases, University of Veterinary Medicine Hannover. Fecal and ruminal fluid samples were directly spread on Schaedler agar plates (Becton Dickinson, Heidelberg, Germany) supplemented with 10 mg/l Vit K1 (Roth, Karlsruhe, Germany) and 5 % bovine blood (WDT, Memsen, Germany), and incubated at 37 °C for 48 h under anaerobic conditions. 25 g of each feed sample were transferred into 225 ml peptone water (Oxoid, Wesel, Germany) and suspended for 20 min in a shaking water bath at room temperature. Afterwards, 1 ml was transferred into liver-bouillon (Oxoid; Wesel, Germany). Additionally, 0.3-0.5 g of each feces and ruminal fluid was transferred into 8 ml liver-bouillon. Spores were selected by heating the bouillon at 80 °C for 10 min in a water bath, followed by incubation at 37 °C for 48 h. Afterwards, one loopfull was spread by dilution-streaking on Reinforced Clostridial Agar (Oxoid, Wesel, Germany) with 12 mg/l Kanamycin (AppliChem, Darmstadt, Germany), 75 mg/l Neomycin (Serva, Heidelberg, Germany) and 10 % bovine blood, as well as on Schaedler agar plates. These plates were then incubated anaerobically at 37 °C for 48 h. Colonies suspected of being *Clostridium* species were transferred to pure cultures. Simultaneously, all isolates were tested for growth under aerobic conditions. Only Gram-positive or -intermediate isolates showing no growth under aerobic conditions were transferred as pure cultures on Columbia agar plates containing sheep blood (Oxoid, Wesel, Germany) to the Institute of Food Quality and Food Safety, University of Veterinary Medicine Hannover for further investigation.

### DNA isolation and amplification of 16S rRNA gene

Colonies from pure cultures were transferred into 180 μl of lysis buffer (lysozyme 20 mg/ml), and incubated for 30 min at 37 °C. DNA was isolated with the DNeasy Blood and Tissue Kit (Qiagen, Darmstadt, Germany) according to the manufacturer’s instructions for Gram-positive bacteria. Elution was carried out with 100 μl of elution buffer. All primers applied in this study were added to the reaction mixture at a concentration of 10 pmol/μl. PCR reaction mixtures for amplification of the 16S rRNA gene consisted of 15 μl mastermix (RedˈyˈGold Mix, Eurogentec, Cologne, Germany), 1 μl primer 16SUNI-L (Table 1), 1 μl primer 16SUNI-R, 0.5 μl MgCl_2_ (50 mM), 7.5 μl nuclease free water, and 5 μl sample DNA. The thermocycling program started with an initial denaturation at 94 °C for 3 min, followed by 35 cycles at 94 °C for 30 s, 55 °C for 30 s and 72 °C for 45 s. Amplification products (5 μl) were loaded onto a 1.5 % agarose gel (Pequlab, Erlangen, Germany), and electrophoresis was performed for 45 min at 10 volt/cm. The remaining 25 μl of amplification product were used for sequencing in both directions (Eurofins genomics, Ebersberg, Germany) with sequencing primers 533 F and 907R (Table 1). All sequences were aligned against two gene databases, NCBI-BLAST (database for 16S ribosomal RNA sequences (Bacteria and Archaea) using the Megablast Tool; http://blast.ncbi.nlm.nih.gov/Blast.cgi?PROGRAM=blastn&PAGE_TYPE=BlastSearch&LINK_LOC=blasthome) and SepsiTest™-BLAST (http://www.sepsitest-blast.de/de/index.html).

**Table 1:** Oligonucleotide primer for 16S rRNA gene amplification and sequencing

Primer	Sequence (5′–3′)	Location^c^
16S rRNA gene amplification primer^a^		
16SUNI-L	AGAGTTTGATCATGGCTCAG	8-27
16SUNI-R	GTGTGACGGGCGGTGTGTAC	1410-1391
16S rRNA gene sequencing primer^b^		
533F	GTGCCAGCAGCCGCGGTAA	514-532
907R	CCGTCAATTCMTTTGAGTTT	925-906

^a^Kuhnert P, Capaul SE, Nicolet J, Frey J. Phylogenetic positions of *Clostridium chauvoei* and *Clostridium septicum* based on 16S rRNA gene sequences. Int J Syst Bacteriol. 1996;46:1174-1176

^b^Petti CA. Detection and identification of microorganisms by gene amplification and sequencing. Clin Infect Dis. 2007;44:1108–1114

^c^Indicating the position of each primer relative to the 16S rRNA gene sequence of *E. coli* J01859

### Toxin-genotyping

All isolates identified as *C. perfringens* were further genotyped using two multiplex PCR assays with primers developed and validated by Baums et al. [17]. Each 25-μl reaction mixture for detection of the four major toxin genes contained 12.5 μl of mastermix (RedˈyˈGold Mix); 0.5 μl of nuclease-free water; 0.5 μl of each primer CPA5L, CPA5R, CPIL, CPIR, ETXL, ETXR; 1 μl of each primer CPB1L, CPB1R; 2 μl MgCl_2_ (50 mM); and 5 μl DNA. PCR assays for detection of the two minor toxin genes *cpe* and *cpb2* were carried out with mixtures containing 12.5 μl of mastermix (Red‘y’Gold Mix), 2.5 μl of nuclease-free water, 1 μl of primer CPEL, 1 μl primer CPER, 0.5 μl primer CPB2L, 0.5 μl primer CPB2R, 2 μl MgCl2, and 5 μl DNA. The amplification program of both assays started with an initial denaturation at 95 °C for 2 min, followed by 35 cycles of 20 s at 94 °C, 20 s at 55 °C and 30 s at 72 °C. For positive controls, in-house collection strains were used (type A: CCUG 1795 T; type B: CCUG 2035; type C: CCUG 2036; type D: CCUG 2037; type E: CCUG 44727). Isolates that were negative for all toxin genes were additionally tested with another primer set for the presence of the *cpa* gene (Table 2) [18]. Reaction mixtures contained 12.5 μl mastermix (RedˈyˈGold Mix), each 0.5 μl of forward and reverse primer, 0.6 μl MgCl_2_ (50 mM), 10.9 μl nuclease-free water, and 5 μl of sample DNA. Amplification was carried out after denaturation for 5 min at 94 °C; with 35 cycles comprising 94 °C for 30 s, 55 °C for 30 s and 72 °C for 30 s; and a final elongation at 72 °C for 10 min. As the *cpb2* primers used in the duplex assay only target the consensus variant of the gene, all isolates were additionally tested with the PCR assay developed by Jost et al. [11], to target both allelic variants, consensus (*cpb2con*) and atypical (*cpb2aty*). Reaction mixtures contained of 12.5 μl mastermix (RedˈyˈGold Mix), 0.5 μl of each primer (CPB2ATYPF, CPB2CONF, CPB2R), 0.6 μl MgCl_2_ (50 mM), 7.9 μl nuclease-free water, and 2.5 μl of sample DNA. Amplification was carried out after denaturation for 5 min at 94 °C; with 35 cycles comprising 94 °C for 30 s, 55 °C for 1 min and 72 °C for 1 min; and a final elongation at 72 °C for 10 min. The positive control for the consensus gene was the above mentioned type A strain (CCUG 1795 T). A field isolate of the in-house collection (LMQS-BP-8160, bovine type A) served as positive control for the atypical *cpb2* variant. Separation of PCR products was performed for all typing assays on 2.0 % agarose gels.

**Table 2:** Oligonucleotide primers for *C. perfringens* toxin gene detection

Primer	Sequence (5′–3′)	Amplificationproduct size [bp]	Primer concentration [mM]	Toxin	Coding toxin gene
Multiplex PCR (major toxin genes)^a^					
CPA5L	AGTCTACGCTTGGGATGGAA	900	200	alpha	*cpa*
CPA5R	TTTCCTGGGTTGTCCATTTC		200		
CPBL	TCCTTTCTTGAGGGAGGATAAA	611	400	beta	*cpb*
CPBR	TGAACCTCCTATTTTGTATCCCA		400		
CPIL	AAACGCATTAAAGCTCACACC	293	200	iota	*iap*
CPIR	CTGCATAACCTGGAATGGCT		200		
CPETXL	TGGGAACTTCGATACAAGCA	396	200	epsilon	*etx*
CPETXR	TTAACTCATCTCCCATAACTGCAC		200		
Duplex PCR (minor toxin genes)^a^					
CPEL	GGGGAACCCTCAGTAGTTTCA	506	400	entero	*cpe*
CPER	ACCAGCTGGATTTGAGTTTAATG		400		
CPB2L	CAAGCAATTGGGGGAGTTTA	200	200	beta-2	*cpb2*
CPB2R	GCAGAATCAGGATTTTGACCA		200		
Singleplex PCR for *cpa* detection^b^					
224	AGGAACTCATGCTATGATTGTAACTCAAGG	775	200	alpha	*cpa*
972inverse	ACCACTAGTTGATATGTAAGCTACTAG		200		
PCR for *cpb2* variants^c^					
CPB2ATYPF	ATTATGTTTAGGAATACAGTTA	304	200	beta-2 atypical	*cpb2aty*
CPB2CONF	CAATTGGGGGAGTTTATCCACAA	741	200	beta-2 consensus	*cpb2con*
CPB2R	CAATACCCTTCACCAAATACTC		200		

^a^Baums CG, Schotte U, Amtsberg G, Goethe R. Diagnostic multiplex PCR for toxin genotyping of *Clostridium perfringens* isolates. Vet Microbiol. 2004;100:11–16

^b^Schlapp T, Blaha I, Bauerfeind R, Wieler LH, Schoepe H, Weiss R, Baljer G. Synthesis and evaluation of a nonradioactive gene probe for the detection of *Clostridium-perfringens* alpha-toxin. Mol Cell Probe. 1995;9:101–109

^c^Jost BH, Billington SJ, Trinh HT, Bueschel DM, Songer JG. Atypical cpb2 genes, encoding beta2-toxin in *Clostridium perfringens* isolates of nonporcine origin. Infect Immun. 2005;73(1):652–6

## Results

In total, 662 isolates of *C. perfringens* were cultured from samples from 129 farms*.* Of these, 442 originated from feces (from 378 individuals without changes in fecal texture and from 16 cows with diarrhea) and 207 from ruminal fluid (from 192 individuals). The 13 feed isolates were obtained from one potato pulp, one pressed pulp silage, and all others from grass silages cut at different times during the year and stored for variable periods, ranging from 8 up to 72 weeks.

With the PCR developed by Baums et al. [17], 17 (2.57 %) of the *C. perfringens* isolates were negative for all six toxin genes, also with repetition. Additional testing with the primers developed by Schlapp et al. [18] lead to a *cpa* positive result for all these 17 isolates. A positive result for the consensus *cpb2* gene was obtained with the duplex PCR for 31 of the 662 isolates [17]. In contrast, with the PCR that was additionally applied to target both allelic variants [11], 94 isolates (including all 31 positive using the duplex PCR assay) and 194 isolates were positive for the consensus and the atypical *cpb2* gene, respectively.

Solely positive for one major toxin gene (*cpa*) and therefore assigned to type A of *C. perfringens* were 660 isolates. Two isolates were assigned to type D (positive for *cpa* and *etx*). Additionally, the consensus and atypical variants of the *cpb2* gene were present in 64 (14.5 %) and 138 (31.2 %) of all isolates from feces, and 30 (14.5 %) and 54 (26.1 %) of all obtained from rumen content, respectively. Of the type A isolates, 5 (0.8 %) were positive for *cpe*. None carried *cpb* or *iap.* Table 3 shows the occurring types in individual samples. This study was not carried out to determine differences between cows with diarrhea and those without. However, after clinical examination when fecal samples were collected, diarrhea was observed in some (*n* = 16) animals. This was considered as potential sign of acute enteric disorder. According to the sampling scheme feces were collected and investigated also from these cows. To exclude study bias toxin typing results of fecal isolates from these animals are given separately in Table 3. No considerable differences were observed between typing results for fecal samples from cows showing diarrhea and those without. Nevertheless, the number of investigated isolates from animals with diarrhea was too small to draw conclusions on differences.

**Table 3:** Genotypes of *C. perfringens* present in feces, rumen content and feed

		*C. perfringens* types										
Sample material		A	Aβ2c	Aβ2a	Ae	A + Aβ2c	A + Aβ2a	A + Ae	Aβ2c + Aβ2a	D	Dβ2c	Total
		*n* = (%)	*n* = (%)	*n* = (%)	*n* = (%)	*n* = (%)	*n* = (%)	*n* = (%)	*n* = (%)	*n* = (%)	*n* = (%)	*n* = (%)
Feces	Diarrhea											
	no	196 (51.9)	54 (14.3)	112 (29.6)	0 (0.0)	3 (0.8)	9 (2.4)	1 (0.3)	1 (0.3)	1 (0.3)	1 (0.3)	378 (100)
	yes	11 (68.8)	1 (6.3)	3 (18.8)	1 (6.3)	0 (0.0)	0 (0.0)	0 (0.0)	0 (0.0)	0 (0.0)	0 (0.0)	16 (100)
Rumen content		107 (55.7)	29 (15.1)	50 (26.0)	3 (1.6)	1 (0.5)	2 (1.0)	0 (0.0)	0 (0.0)	0 (0.0)	0 (0.0)	192 (100)
Feed		10 (76.9)	1 (7.7)	2 (15.4)	0 (0.0)	0 (0.0)	0 (0.0)	0 (0.0)	0 (0.0)	0 (0.0)	0 (0.0)	13 (100)

type A = *cpa* ^+^; type Aβ2c = *cpa* ^+^ + *cpb2con* ^+^; type Aβ2a = *cpa* ^+^ + *cpb2aty* ^+^; type Ae = *cpa* ^+^ + *cpe* ^+^; type D = *cpa* ^+^ + *etx* ^+^; type Dβ2c = *cpa* ^+^ + *etx* ^+^ + *cpb2con* ^+^

Isolation of more than one genotype from single samples is indicated by listing each genotype separated by a “+”

## Discussion

Studies on the occurring types of *C. perfringens* in mature dairy cattle including all toxin genes investigated within this study are rare. For the presence of the *cpb2* and the *cpe* gene broad differences were reported for cattle [19–22]. Therefore, 662 *C. perfringens* isolates from dairy cattle were toxin genotyped to gain further insight into the presence of its different types on dairy farms.

Mainly *C. perfringens* type A was detected. This is the most frequently isolated type in samples of human, animal or environmental origin [3]. Only two isolates (0.3 %) of all were designated to be *C. perfringens* type D (*cpa* and *etx* positive), which is known to cause enterotoxaemia in small ruminants. Additionally, reports exist which assume that it plays a role in disease in calves [5, 23, 24]. Both investigated type D positive cows showed no signs of acute disorders, similar to those described for disease in small ruminants affected by type D. Within this study, none of the investigated isolates carried genes for the beta or the iota toxin found in types B, C and/or E. Though, for example, iota toxin gene positive *C. perfringens* (type E) were previously detected in healthy calves as well as in those suffering from diarrhea [25].

The beta-2 toxin is known to be associated with enteritis in piglets and a causative role of it in enteric disease of calves was assumed [8, 9, 26]. Of all isolates cultured in this study, 202 (45.7 %) and 82 (39.6 %) obtained from feces and rumen content harbored the *cpb2* gene, respectively. A similar rate (46 %) was found by Lebrun et al. who investigated isolates obtained from 14 healthy calves [27]. A screening conducted in North America for *cpb2* in field isolates obtained from clinical laboratories detected the gene in 12.8 % of all isolates from cattle (including isolates from animals of all ages with and without enteric disease) [19]. Within that study a much higher rate of 47.3 % in isolates from calves suffering from enteric disease was found. This was mainly due to the high detection rate in type E isolates (97.3 %). A previous study conducted in Belgium, detected this gene in around 30 % of all isolates that were obtained equally from around 60 % of all healthy and diarrheic calves [25]. Gurjar et al. investigated feces of cows from seven dairy farms and found the toxin gene in 68 % of all *C. perfringens* positive fecal samples [20]. Within the present study we distinguished between both allelic variants of *cpb2.* The so called atypical *cpb2* was detected as well in feces as in rumen content about twice as often as the consensus variant. Previous studies found that in porcine isolates the consensus variant occurs more frequently [11]. In contrast, in isolates of non-porcine origin the atypical variant is more common, whereby variations exist between different animal species [11, 28]. Until now mainly small numbers of isolates obtained from cattle have been investigated discriminating between the two allelic variants of this gene. Jost et al. tested 24 isolates from cattle and found the atypical *cpb2* in seven of ten type A, both type C and 12 of 12 type E strains [11]. Kircanski et al. tested bovine isolates and found the consensus and the atypical variant in 1 and 5 of 11 isolates, respectively [28]. The study from Lebrun et al. focusing on calves suffering from diarrhea detected in these solely the consensus variant [27]. Also, in isolates obtained from eight healthy calves the consensus variant (67.5 %) was detected more frequently than the atypical allele (32.5 %). One larger study was carried out on 218 bovine *C. perfringens* isolates from healthy cattle and animals suffering from acute enteric disease [29]. Deviating from the present study, Schlegel et al. found in 13 % of all isolates the atypical variant, but in none the consensus *cpb2.* Despite the varying detection frequencies, our results and those of previous studies demonstrate that both allelic variants occur frequently in cattle, whereby the atypical *cpb2* seems to dominate.

Human enteric diseases, due to *C. perfringens*, are mainly caused by strains producing the enterotoxin [4]. These food poisoning strains are among those pathogens most frequently associated with foodborne illnesses [12, 30]. *cpe* was only detected in five (0.8 %) of all investigated isolates in the present study indicating that dairy cows are no primary source for these food poisoning strains. Kukier et al. reported similar findings of 0.34 % *cpe* positive isolates from bovine fecal samples [21]. A higher rate was reported by Gurjar et al. [20], who found the enterotoxin gene in 4.6 % of all PCR positive fecal samples from dairy cows in Pennsylvania, USA. Miwa et al. even reported a rate of 26 % *cpe* positive feces of healthy slaughter cattle in Japan using nested PCR [22]. Most studies on the epidemiology of enterotoxigenic *C. perfringens* in farm animals did not recover isolates, and therefore Lindstroem et al. presumed that maybe unspecific amplification lead to the finding of high rates [12, 22]. In contrast, within the present study only cultured isolates confirmed to be *C. perfringens* were tested for the presence of *cpe*. Differences between studies may also display the reported divergence in detection of *C. perfringens* toxin types in sample material of different geographical locations, as suggested for *cpb2* positive isolates [10].

*C. perfringens* was found not only ubiquitous in feces but also in rumen content of cows within this study. This is to the authors knowledge the first investigation, that toxinotyped such a large number of *C. perfringens* isolates (*n* = 207) obtained from rumen content of cows. From this matrix only type A was cultured. A few isolates were tested positive for *cpe* and a larger amount for consensus or atypical *cpb2*.

Due to the culture conditions, not specifically selective for *C. perfringens*, isolates were obtained from fewer samples than it could have been expected with a more selective method. Nonetheless, most previous studies tested field isolates (often from animals with acute enteric disease) or isolates from calves. In contrast, this study examined a large number of isolates obtained from mature dairy cattle. Diarrhea was considered within this study as a sign of acute enteric disease. Thus, typing results of fecal samples of these animals are presented separately (Table 3), although the study was not carried out to determine differences between cows with diarrhea and those without. No considerable differences were detected between fecal samples of animals showing diarrhea and those without.

With the PCR developed and validated by Baums et al. [17], 17 (2.57 %) of all *C. perfringens,* that had previously been identified on the basis of 16S rRNA gene sequencing, gave negative results for the *cpa* gene. As it is known to be chromosomally coded and highly conserved, all *C. perfringens* isolates are expected to carry this gene [1]. Therefore, an additional PCR was applied using previously published primers to test the negative isolates for the presence of the *cpa* gene. With the primers developed by Schlapp et al. [18] all isolates that were negative with the first primer set gave positive results. Also other authors found small numbers of presumed *cpa* negative isolates of animal origin [29, 31]. When tested with another PCR, these isolates of those previous studies may also give positive results. A possible reason for these negative PCR outcomes may be nucleotide variations in the primer binding region [22]. *C. perfringens* confirmation is often done by detection of the *cpa* gene. Considering that around 2.6 % of the investigated isolates gave false negative results using one PCR assay, this point needs attention. The primer set for *cpb2* detection used in the duplex assay [17] only detects the consensus variant of the *cpb2* gene. Thus, we additionally applied a PCR that distinguishes between the consensus and the atypical variant. We found differing results between the two primer sets targeting the consensus variant of the *cpb2* gene published by Baums et al. [17] and Jost et al. [11]. With the primers used in the duplex assay 31 isolates were tested positive for the consensus *cpb2*, whereas with the more recent primer set described by Jost et al. 94 isolates (including all 31 isolates positive with the first assay) gave a positive result. A previous study investigated the genetic diversity of *cpb2* genes and found for the consensus variant sequence similarities between 93.3 and 100 % [32]. Thus, apart from the genetically low relatedness between the consensus and the atypical variant also within the consensus allele nucleotide variations exist. This could explain the differing results obtained with both assays.

## Conclusion

The diversity of *C. perfringens* types was low as mainly type A was cultivated. Of all isolates from feces and rumen content 202 (45.7 %) and 82 (40.6 %) harbored one variant of the *cpb2* gene, respectively. The atypical allele occurred about twice as often as the consensus variant. The enterotoxin gene was present in only 0.8 % of all isolates. Thus, the present study indicates that dairy cows are no primary source for potentially human pathogenic enterotoxin gene positive strains.
